# Effect of Couple Stress and Mass Transpiration on Ternary Hybrid Nanoliquid over a Stretching/Shrinking Sheet with Heat Transfer

**DOI:** 10.3390/mi13101694

**Published:** 2022-10-09

**Authors:** Kolkar Nanjappa Sneha, Gadabanahalli Puttasiddappa Vanitha, Ulavathi Shettar Mahabaleshwar, David Laroze

**Affiliations:** 1 Department of Mathematics, Davangere University, Shivagangothri, Davangere 577007, India; 2 Instituto de Alta Investigación, CEDENNA, Universidad de Tarapacá, Casilla 7D, Arica 1000000, Chile

**Keywords:** couple stress, thermal radiation, ternary hybrid nanofluid, stagnation point, heat transfer, Biot number

## Abstract

The present article describes the unsteady flow of a couple stress via a ternary hybrid nanofluid on a stretching surface with porous media. The nanofluid exhibits important properties for increasing heat transmission, and it is widely used in manufacturing and industrial applications. The basic similarity equations have been discovered to accommodate both stretching/shrinking surfaces. Ternary hybrid nanofluid is a colloidal combination of three types of microspheres: Al_2_O_3_, single wall carbon nanotubes, and graphene. For investigating spherical, cylindrical, and platelet nanoparticles, the governing partial differential equations are converted into ordinary differential equations, expending appropriate transformations. The analytical solution can then be carried out using various forms of nanoparticles, such as spherical, cylindrical, and platelet, to obtain the solution domain. Heat transfer is used in an electrically conducting fluid and also including thermal radiation, as calculated with the Biot number. The focus of the present effort is the evaluation of the flow of ternary hybrid nanofluid over a porous media via thermal radiation, with couple stress, using an analytical process. For various physical parameters, the velocity and temperature conditions are shown graphically.

## 1. Introduction

The authors investigated the stretching sheet problem by examining its importance and applicability in industrial processes such as polymer extrusion, paper production, metal cooling, and glass blowing, among others [1]. The pioneering work on the surface stretching problem is by Sakiadis [2,3]. Crane [4] takes up the problem of the stretched surface, where the Newtonian flow is linearly changing from the slit. Later, several studies were conducted on the stretching sheet problem with several boundary conditions. In view of stream and water surface is investigated by Gu et al. [5]. The thermal conductivity and dynamic viscosity have calculated as these possibilities, the impact of mass transfer and energy properties concerning the air by Esfe et al. [6] using a hybrid nanofluid. Bhattacharyya et al. [7] studied the properties of hydrodynamic heat transport. Chamkha et al. [8] found that heat transfer across the dynamics of a hybrid nanofluid is higher than that of an alumina-water nanofluid, but lower than that of a copper-water nanofluid. Mahdavi et al. [9] investigated a fluid flow and heat transfer analysis of nanofluid jet cooling on a hot surface with various roughnesses. MHD nanofluid flow with suction, as well as magnetohydrodynamics a nanofluid flow in natural convection with a porous term, was studied by Benos et al. [10]. Besides these mixture, hybrid nanofluids are defined as fluids containing two types of nanoparticles. A hybrid nanofluid is a specific type of fluid that has superior thermal conductivity to either nanofluids or base fluids. 

Anusha et al. [11,12] investigated a hybrid nanofluid that included both transpiration and radiation. Sharifpur et al. [13] conducted an experimental investigation and developed a model for evaluating the thermal conductivity of α-Al_2_O_3_-glycerol nanofluids. In industrial settings, such as in the acceleration of sheets, where liquid cooling is crucial to achieving the desired result, the temperature distribution impacted by thermal radiation is highly significant. This type of flow problem can be seen in engineering applications such as extrusion operations, metal spinning, dye casting of metals, and polymer manufacture, where the maximum temperature and difference prediction should be controlled. This study has attracted the interest of many researchers due to the numerous exciting industrial and everyday applications of shear-thinning fluids. Wall paint, printing ink, nail polish, whipped cream, ketchup, and engine oil are a few examples of these. Shear thinning fluid, also known as pseudo-plastic fluid, is thought to exhibit characteristics of both plastic and Newtonian fluid. The more tension that is applied to a shear-thinning fluid, the more freely the fluid flows. This quality makes it a beneficial trait for products such as paint, oils, and cream [14,15,16].

According to a study by Zhang et al. [17] on thermal diffusivity and thermal conductivity, if a colloidal mixture of various nanofluids is generated by water conveying cylindrical and spherical nanoparticles, the normalized thermal conductivity of the cylindrical nanoparticles increases. Furthermore, water transporting CNT nanoparticles has a far higher thermal conductivity than nanofluids carrying spherical nanoparticles. Wakif et al. [18] explored the internal heating of spatially homogeneous and non-uniform nanofluids. Narayana et al. [19] studied an unstable MHD nanofluid subjected to non-uniform heat generation and absorption. Many equations have been proposed for to nanofluid fluid movement across porous media. The formal structure of issues regarding a porous medium was suggested by Darcy and Brinkmann [20,21]. Anuar et al. [22] studied the heat transfer of carbon nanotubes over an exponentially stretching/shrinking sheet using suction and the slip effect. Anwar et al. [23] investigated the unsteady MHD flow of Jeffery fluid flow using wall velocity and Newtonian fluid. The fluid flow and heat transfer of carbon nanotubes, along with a flat plate with a Navier slip boundary, were investigated by Khan et al. [24]. Shalini et al. [25] studied the unsteady MHD chemically reacting mixed convection nanofluid flow past an inclined pour stretching sheet, using the slip effect, as well as a variable thermal radiation and heat source The MHD flow of a Jeffery fluid with mixed convection on a porous medium was investigated by Yana et al. [26] using a radiative heat flux stretching/shrinking surface. Stokes is recognized for developing the concepts and equations for pair stress fluid flows, which is another important point to make. The governing equations are provided for an incompressible pair stress fluid flow with conservative body forces. Some insights regarding these ideas can be found in refs. [27,28]. 

The current work is an examination of ternary hybrid nanofluid flow under the major effects of a couple stress parameters, radiation, and transpiration, as seen in the above-mentioned studies. Constitutive law is one of the main ideas at work here; it is the relationship between the forces imposed on that substance and the resulting deformation at a microscopic level. It also has to do with how much strain a body is experiencing in relation to how much stress the material is under. A porous medium is used to stretch the fluid. Researchers are interested in conducting a stretching sheet experiment with a porous medium because fluid flow with a porous medium has several industrial applications. In addition, the current situation is an example of a couple stress with a porous media challenge. By using the appropriate similarity variables, the governing equations of the supplied PDEs are mapped into ODEs. The solution domain is then obtained by solving the ODEs analytically. The solution is achieved with the use of the incomplete gamma function regarding the heat transfer, which is induced by a stretching sheet with a linear velocity variation from the slit, with mass suction included. Throughout the equation, solid volume fractions of the three distinct nanoparticle structures are employed. In fact, there are few studies in the literature that cover so many aspects of this technique. These insights could be used in the development of industrial procedures for the creation of the best product. The novelty of the present work explains the flow of couple stress fluids, especially unsteady flow, with three types of different nanoparticles; thus, we extract the results on the basis of the analytical method. This work motivated researchers to conduct more studies on stretching sheet problems with the porous medium in the presence of ternary nanofluids, since there are many equations and derivations available to explain the porous medium and thermal radiation. 

## 2. Physical Model and Formulations

**Figure 1:**
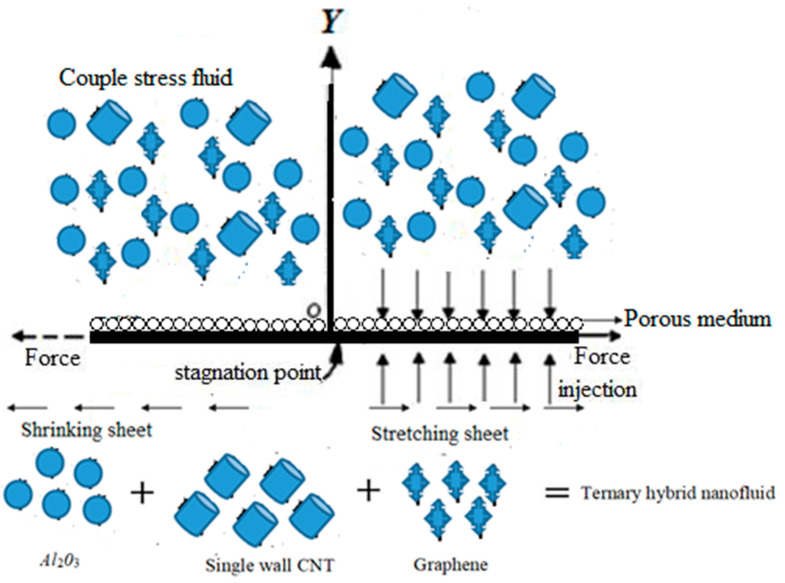
Physical model of the ternary hybrid nanofluid.

The current study considers a couple stress fluid flow over a porous stretching sheet under the influence of radiation and mass transpiration. In the present work, we use non-Newtonian coupling. Moreover, it should be noted that the water containing spherical aluminum oxide, cylindrical single wall CNT, and platelet graphene nanoparticles, as shown in Figure 1, is transported. Table 1 shows the thermal characteristics of these three nanofluids. The following formula is used to calculate the unstable flow stream velocity far enough from the sheet $U_{\infty }=-\frac{U_{0}x}{1-\gamma t}$, suppose that $U_{w}$ and $V_{w}$ are the wall movement speeds, so the standard absorption speed is taken into account. The exterior free stream velocity at the wall is calculated as follows $U_{w}=\frac{\lambda U_{0}x}{1-\gamma t}$, where, λ represents the constant, and λ>0 the sustaining flow, if λ<0 be in conflict with the flow λ=0.

Under these considerations, the continuity of the Navier–Stokes equation can be written as:

(1) $$\frac{\partial u}{\partial x}+\frac{\partial v}{\partial y}=0$$

(2) $$\rho_{tnf}\left(\frac{\partial u}{\partial t}+u\frac{\partial u}{\partial x}+v\frac{\partial u}{\partial y}\right)=-\frac{\partial P}{\partial x}+\mu_{tnf}\left(\frac{\partial^{2}u}{\partial x^{2}}+\frac{\partial^{2}u}{\partial y^{2}}\right)-\frac{\mu_{tnf}}{K}\left(u_{\infty }-u\right)-\eta_{0}\frac{\partial^{4}u}{\partial y^{4}}\;$$

(3) $$\rho_{tnf}\left(\frac{\partial v}{\partial t}+u\frac{\partial v}{\partial x}+v\frac{\partial v}{\partial y}\right)=-\frac{\partial P}{\partial y}+\mu_{tnf}\left(\frac{\partial^{2}v}{\partial x^{2}}+\frac{\partial^{2}v}{\partial y^{2}}\right)\;$$

(4) $${\left(\rho C_{P}\right)}_{tnf}\left(\frac{\partial T}{\partial t}+u\frac{\partial T}{\partial x}+v\frac{\partial T}{\partial y}\right)=\kappa_{tnf}\left(\frac{\partial^{2}T}{\partial x^{2}}+\frac{\partial^{2}T}{\partial y^{2}}\right)-\frac{\partial q_{r}}{\partial y}$$

Note that Equation (2) includes extra terms that provide a model beyond the standard Newtonian fluid, which are common for non-Newtonian fluids. In particular, the biharmonic term proportional to $\eta_{0}$ represents the connection between materials considering the couple stress. In addition, the imposed boundary conditions are as follows:

(5) $$\begin{array}{l} u\left(x,0,t\right)=\frac{\lambda U_{0}x}{1-\gamma t},\;\;\;\;\;\;\;\;\;\;v\left(x,0,t\right)=V_{w}\left(x,t\right),\;\;\;\;\;\;-\kappa_{f}\frac{\partial T}{\partial y}\;=\;h(T_{w}-T_{\infty })\;\;\;at\;\;\;\;y=0,\\ \;\;\;u\left(x,\infty ,t\right)=-\frac{U_{0}x}{1-\gamma t},\;\;\;\;\;\;\;\;\;\;\theta \left(\infty \right)=\;0.\;\;\;\;\;\;\;\;\;\;\;\;\;\;\;\;\;\;\;\;\;\;\;\;\;\;\;\;\;\;\;\;\;\;\;\;as\;\;y\to \infty . \end{array}$$

The dimensionless variables are easier to locate via the similarity transformation.

(6) $$\psi \left(x,y,t\right)=x\sqrt{\frac{\nu_{f}U_{0}}{1-\gamma t}}f\left(\eta \right),\;\;\;\;\;\theta \left(\eta \right)\;=\;\frac{\left(T-T_{w}\right)}{\left(T_{w}-T_{\infty }\right)},\;\;\;\eta =y\sqrt{\frac{U_{0}}{\nu_{f}\left(1-\gamma t\right)}},\;\;\;\;\;\;\;\;\;\;$$

The adopted similarity functions are as below:

(7) $$u=\frac{U_{0}x}{1-\gamma t}f_{\eta }\left(\eta \right),\;\;\;\;\;\;\;\;\;\;\;\;\;\;\;\;\;v=-\sqrt{\frac{\nu_{f}U_{0}}{1-\gamma t}}\;f\left(\eta \right).$$

As a result, the velocity of the wall transpiration increases $v_{w}\left(x,0,t\right)=\sqrt{\frac{U_{0}\nu_{f}}{1-\gamma t}}f\left(0\right)$.

The boundary layer hypothesis is used in the momentum and energy equations, which are two-dimensional equations. The thermal boundary conditions for two altered situations will be explained advanced. By conjecturing two similarity equations.

(8) $$\Lambda f^{v}-A_{1}\;f^{\prime \prime \prime }-A_{2}\left[f\;f^{\prime \prime }-f^{\prime 2}+1-\beta \left(f+\frac{1}{2}\eta f^{\prime \prime }-1\right)\right]-A_{1}\;Da^{-1}\left(1-f^{\prime }\right)=0,$$

(9) $$\left(A_{4}+N_{R}\right)\theta^{\prime \prime }+c_{3}Pr\left(f-\frac{\beta \eta }{2}\right)\theta^{\prime }=0.$$

The modified boundary conditions are:

(10) $$\begin{array}{l} f^{\prime }=\lambda ,\;\;\;f\left(0\right)=V_{C},\;\;\;\;\;\theta^{\prime }\;=\;\;-Bi\left(1-\theta \left(0\right)\right)\;\;\;\;\;as\;\;\;y=0,\;\;\;\;\;\;\;\;\;\;\;\;\\ f^{\prime }=-1,\theta \left(\infty \right)\to 0\;\;\;\;\;\;\;\;\;\;\;\;\;\;\;\;\;\;\;\;\;\;\;\;\;\;\;\;\;\;\;\;\;\;\;at\;\;y\to \infty . \end{array}$$

The various physical parameters are as follows:

$\Lambda =\frac{U_{0}\eta_{0}}{(1-\gamma t)\nu_{f}\rho_{f}}$ Couple stress, $Pr\;=\;\frac{\nu_{f}}{\alpha_{f}},$ Prandtl number, $Da^{-1}=\frac{\mu_{f}\left(1-\gamma t\right)}{K\rho_{f}U_{0}}$ Porous media, $N_{R}=\frac{16\sigma^{*}T_{\infty }{}^{3}}{3k^{*}\kappa_{f}}$ Thermal radiation [29,30], $\lambda \;=\;\;-\;\frac{U_{w}}{U_{\infty }},$ λ> 0 Stretching sheet λ< 0 is shrinking sheet parameters.

The quantities of ternary hybrid nanofluid are given below:

(11) $$\begin{array}{l} \phi =\phi_{1}+\phi_{2}+\phi_{3}\\ \mu_{tnf}=\frac{\mu_{nf_{1}}\phi_{1}+\mu_{nf_{2}}\phi_{2}+\mu_{nf_{3}}\phi_{3}}{\phi }\\ \kappa_{tnf}=\frac{\kappa_{nf_{1}}\phi_{1}+\kappa_{nf_{2}}\phi_{2}+\kappa_{nf_{3}}\phi_{3}}{\phi }\\ \;\rho_{tnf}=\left(1-\phi_{1}-\phi_{2}-\phi_{3}\right)\rho_{bf}+\phi_{1}\rho_{sp_{1}}+\phi_{2}\rho_{sp_{2}}+\phi_{3}\rho_{sp_{3}}\\ {\left(\rho C_{P}\right)}_{tnf}=\left(1-\phi_{1}-\phi_{2}-\phi_{3}\right){\left(\rho C_{P}\right)}_{bf}+\phi_{1}{\left(\rho C_{P}\right)}_{sp_{1}}+\phi_{2}{\left(\rho C_{P}\right)}_{sp_{2}}+\phi_{3}{\left(\rho C_{P}\right)}_{sp_{3}} \end{array}$$

Suganthi and Rajan et al. [31] discovered that the motion of solid particles in solid–liquid dispersion is dependent on particle form. Non-spherical particles have more motion and fluid flow resistance than spherical particles.

The number of spherical nanoparticles is determined by:

(12) $$\begin{array}{l} B_{1}=\frac{\mu_{nf_{1}}}{\mu_{bf}}=1+2.5\phi +6.2\phi^{2}\\ B_{4}=\frac{\kappa_{nf_{1}}}{\kappa_{bf}}=\frac{\kappa_{sp_{1}}+2\kappa_{bf}-2\phi \left(\kappa_{bf}-\kappa_{sp_{1}}\right)}{\kappa_{sp_{1}}+2\kappa_{bf}+\phi \left(\kappa_{bf}-\kappa_{sp_{1}}\right)} \end{array}$$

the quantities of cylindrical nanoparticles are given by:

(13) $$\begin{array}{l} B_{2}=\frac{\mu_{nf_{2}}}{\mu_{bf}}=1+3.5\phi +904.4\phi^{2}\\ B_{5}=\frac{\kappa_{nf_{2}}}{\kappa_{bf}}=\frac{\kappa_{sp_{2}}+3.9\kappa_{bf}-3.9\phi \left(\kappa_{bf}-\kappa_{sp_{2}}\right)}{\kappa_{sp_{2}}+3.9\kappa_{bf}+\phi \left(\kappa_{bf}-\kappa_{sp_{2}}\right)} \end{array}$$

the quantities of platelet nanoparticles are given by

(14) $$\begin{array}{l} B_{3}=\frac{\mu_{nf_{3}}}{\mu_{bf}}=1+37.1\phi +612.6\phi^{2}\\ B_{5}=\frac{\kappa_{nf_{3}}}{\kappa_{bf}}=\frac{\kappa_{sp_{3}}+4.7\kappa_{bf}-4.7\phi \left(\kappa_{bf}-\kappa_{sp_{3}}\right)}{\kappa_{sp_{3}}+4.7\kappa_{bf}+\phi \left(\kappa_{bf}-\kappa_{sp_{3}}\right)} \end{array}$$

To make further analysis easier, the following substitutions can be made, as follows:

(15) $$\begin{array}{l} A_{1}=B_{1}\phi_{1}+B_{2}\phi_{2}+B_{3}\phi_{3}\\ A_{2}=1-\phi_{1}-\phi_{2}-\phi_{3}+\phi_{1}\frac{\rho_{sp_{1}}}{\rho_{bf}}+\phi_{2}\frac{\rho_{sp_{2}}}{\rho_{bf}}+\phi_{3}\frac{\rho_{sp_{3}}}{\rho_{bf}}\\ A_{3}=B_{4}\phi_{1}+B_{5}\phi_{2}+B_{6}\phi_{3}\\ A_{4}=1-\phi_{1}-\phi_{2}-\phi_{3}+\phi_{1}\frac{{\left(\rho C_{p}\right)}_{sp_{1}}}{{\left(\rho C_{p}\right)}_{bf}}+\phi_{2}\frac{{\left(\rho C_{p}\right)}_{sp_{2}}}{{\left(\rho C_{p}\right)}_{bf}}+\phi_{3}\frac{{\left(\rho C_{p}\right)}_{sp_{3}}}{{\left(\rho C_{p}\right)}_{bf}} \end{array}$$

### 2.1. Solution of Pressure Gradient

Its assets show that the pressure gradient $\frac{\partial P}{\partial y}$ is a consequence of *t* and *y*, as determined by the velocity elements, and Equation (4) is sovereign by *x*, namely, $\frac{\partial P}{\partial y}=F(t,y)$ and P = ∫F(t,y) dy + G(t,x) somewhere G(t,x) denotes the integration constant. $\frac{\partial P}{\partial x}\;=\;\frac{\partial G\left(t,x\right)}{\partial x}$, which would be independent of *y* and remains equal inside the boundary layer and could be deduced in the free stream. Compute the *x*-momentum to the free stream with $u\;=\;U_{\infty }\;\text{and}\;\partial p/\partial x$

(16) $$-\frac{1}{\rho }\frac{\partial P}{\partial x}=\left(1-\beta \right)\frac{\varepsilon_{2}U_{0}{}^{2}x}{{\left(1-\gamma t\right)}^{2}},\;$$

The velocity acceleration/deceleration feature is shown by β, where $\beta =\frac{\gamma }{U_{0}}$ is the unsteadiness parameter. When β > 0, wall velocities accelerate, β < 0, the wall decelerates, β = 0 steady state, and $p_{0}$ is integration constant.

(17) $$\frac{1}{\rho_{f}}\left(P_{0}-P\right)=\frac{A_{2}}{2}\left(1-\beta \right)\frac{U_{0}{}^{2}x^{2}}{{\left(1-\gamma t\right)}^{2}}+A_{2}\int \frac{\partial v}{\partial t}\;dy+A_{2}\frac{v^{2}}{2}-A_{1}\nu_{f}\frac{\partial v}{\partial y},$$

### 2.2. Solution of Momentum and Energy Equation

In order to determine the momentum equation, an additional similarity variable F(η)=f(η)+η is brought into the system, and the resultant governing equation is:

(18) $$-\Lambda F^{v}+A_{1}F^{\prime \prime \prime }+A_{2}\left(FF^{\prime \prime }-4F^{\prime }-F^{\prime 2}\right)+A_{1}Da^{-1}F^{\prime }=0$$

In addition, the specified governing boundary conditions are transformed into:

(19) $$\begin{array}{l} F\left(0\right)=V_{C},\;\;\;\;F^{\prime }=\lambda +1,\;\;\;at\;\;\eta =0,\\ F^{\prime }=0,\;\;\;\;\;\;\;\;\;\;\;\;\;\;\;\;\;\;\;\;\;\;\;\;\;\;as\;\;\eta \to \infty . \end{array}$$

In the boundary conditions, we get solution $p+q=V_{C}$, $q=\;-\frac{\left(\lambda -1\right)}{\alpha }$ and:

(20) $$F\left(\eta \right)=V_{C}+\frac{\lambda -1}{\alpha }\left[1-\exp\left(-\alpha \eta \right)\right]$$

Apply the assumed solution to Equation (20) into Equation (18) to obtain the exponent α value as:

(21) $$\Lambda \alpha^{4}-A_{1}\alpha^{2}+A_{2}V_{C}\alpha +\left(A_{2}\lambda +3A_{2}-A_{1}Da^{-1}\right)=0$$

Then we obtain the solution:

(22) $$f\left(\eta \right)=\eta +V_{C}+\frac{\lambda -1}{\alpha }\left[1-\exp\left(-\alpha \eta \right)\right]$$

### 2.3. Solution of Heat Transfer

The heat transfer equation can be modified through the use of a significant non-dimensional temperature as:

(23) $$\;\theta \left(\eta \right)\;\;\;=\;\;\;\;\frac{\left(T-T_{w}\right)}{\left(T_{w}-T_{\infty }\right)}$$

We propose new variable ξ that satisfies the correlation coefficients to determine the exact solution of the temperature Equation (9) for the stretching/shrinking sheet.

(24) $$\xi =\left(1-\lambda \right)\frac{Pr}{\alpha^{2}}e^{-\alpha \eta }.$$

Substituting F(η) and Equation (24) in Equation (9) yields the subsequent:

(25) $$\left(A_{4}+N_{R}\right)\;\xi \theta^{\prime \prime }+\left(\left(A_{4}+N_{R}\right)-A_{3}Pr\left(1+\frac{4A_{2}+A_{1}\Gamma }{\alpha^{2}}\right)+A_{3}\xi \right)\theta^{\prime }=0$$

The boundary conditions reduces to:

(26) $$\left(1-\lambda \right)\frac{Pr}{\alpha }\theta^{\prime }\left(\left(1-\lambda \right)\frac{Pr}{\alpha^{2}}\right)\;=\;-Bi\;\left(1-\theta \left(\left(1-\lambda \right)\frac{Pr}{\alpha^{2}}\right)\right),\;\;\theta \left(\infty \right)\;=0.$$

The singular point of the transformation (25) is ξ=0. As a result, the Frobenius method is used to analyze the power solutions of Equation (25):

(27) $$\theta \left(\xi \right)=\sum_{r=0}^{\infty }a_{r}\;\xi^{n+r}.$$

After derivative of the first and second order Equation (27) *w*. *r*. *t*. ξ and upon combining the above-mentioned $\theta^{\prime }\;\text{and}\;\;\theta^{\prime \prime }$ expressions, Equation (25) is defined as:

(28) $$\xi \;\left(A_{4}+N_{R}\right)\;\;\sum_{r=0}^{\infty }\left(n+r\right)\;\left(n+r-1\right)a_{r}\;\xi^{n+r-2}+\;\;\left(\left(A_{4}+N_{R}\right)-A_{3}Pr\left(1+\frac{4}{\alpha^{2}}\right)+A_{3}\xi \right)\;\;\sum_{r=0}^{\infty }\left(n+r\right)\;a_{r}\;\xi^{n+r-1}\;=0\;.$$

here applying the recurrence relation and the following values of $n\;=0,\;\;n=\;1-\frac{k_{1}}{k_{2}}$ respectively:

(29) $$a_{r}\;=\;-\frac{\varepsilon_{3}\left(n+r-1\right)}{\left(n+r\right)\left[c_{2}\left(n+r-1\right)+c_{1}\right]}\;a_{r-1}$$

where:

$$k_{2}\;=\;\left(A_{4}+N_{R}\right),\;\;\text{and}\;k_{1}\;=\;\left(A_{4}+N_{R}\right)-A_{3}\;Pr\left(-\Lambda \alpha^{2}+A_{1}+\frac{4A_{2}+A_{1}\Gamma }{\alpha^{2}A_{2}}\right)$$

The solution of Equation (28) is simplified as follows using this recurrence relation, as well as the variables of *n*.

(30) $$\theta \left(\xi \right)=C_{1}C_{0}+C_{2}C_{0}\left(1-\frac{k_{1}}{k_{2}}\right)\;{\left(-A_{3}\right)}^{-\left(1-\frac{k_{1}}{k_{2}}\right)}\left[\Gamma \left(1-\frac{k_{1}}{k_{2}},\;0\right)-\Gamma \left(1-\frac{k_{1}}{k_{2}},\;-\xi A_{3}\right)\right].$$

Finding the values of $C_{1}$ and $C_{2}$, we get:

$$C_{1}=0,$$

$$\begin{array}{l} C_{2}=\frac{Bi}{c_{0}\left(1-\frac{k_{1}}{k_{2}}\right){\left(-A_{3}\right)}^{\frac{k_{1}}{k_{2}}-1}}\{\alpha {\left(-\left(\lambda -1\right)A_{3}\frac{Pr}{\alpha^{2}}\right)}^{1-\frac{k_{1}}{k_{2}}}\exp\left(\left(\lambda -1\right)A_{3}\frac{Pr}{\alpha^{2}}\right)+\\ \;\;\;\;\;\;\;\;\;\;\;\;\;\;\;\;\;\;\;\;\;\;\;\;\;\;\;\;\;\;\;\;\;\;\;\;\;\;\;\;\;\;\;\;\;\;\;\;\;\;\;\;\;\;\;\;\;{Bi\;\Gamma \left(1-\frac{k_{1}}{k_{2}},0\right)-Bi\;\Gamma \left(1-\frac{k_{1}}{k_{2}},-\left(\lambda -1\right)A_{3}\frac{Pr}{\alpha^{2}}\right)\}}^{-1} \end{array}$$

The conjecture in terms of η is given by substituting the $C_{1}$ and $C_{2}$ values in Equation (30) and the incomplete gamma function defined.

(31) $$\theta \left(\eta \right)=\frac{Bi\;\Gamma \left(1-\frac{k_{1}}{k_{2}},0\right)-Bi\;\Gamma \left(1-\frac{k_{1}}{k_{2}},-\left(\lambda -1\right)A_{3}\frac{Pr}{\alpha^{2}\left(A_{4}+N_{R}\right)}e^{-\alpha \eta }\right)}{\{\alpha {\left(-\left(\lambda -1\right)A_{3}\frac{Pr}{\alpha^{2}}\right)}^{1-\frac{k_{1}}{k_{2}}}\exp\left(\left(\lambda -1\right)A_{3}\frac{Pr}{\alpha^{2}}\right)+Bi\;\Gamma \left(1-\frac{k_{1}}{k_{2}},0\right)-Bi\Gamma \left(1-\frac{k_{1}}{k_{2}},-\frac{\left(\lambda -1\right)}{\left(A_{4}+N_{R}\right)}A_{3}\frac{Pr}{\alpha^{2}}\right)\}}$$

## 3. Results and Discussion

The current study examines a ternary hybrid nanofluid flow towards a porous stretching surface using water as the base fluid. To analyze the current situation analytically, the volume fraction of aluminum oxide, single wall CNT, and graphene nanoparticles are all included in the equation. The current problem’s ODEs were derived from controlling PDEs with similar variables that were solved analytically to provide the solution domain. The solution domain can be used to investigate solution domain, axial velocity, and heat transfer. The results of the current effort can then be examined using various physical characteristics and graphical arrangements. In this unsteady case, the porous medium parameter and thermal radiation play a major role in the stretching sheet problems. The fluid flow due to the porous medium is applicable in many industrial applications, such as the geophysical and allied areas. The results of three different nanoparticles can be examined in the present analysis; these ternary nanoparticles exhibit better thermal efficiency than that of normal nanofluids. Moreover, the couple stress fluid parameter is used to characterize the non-Newtonian fluid behavior.

**Figure 2:**
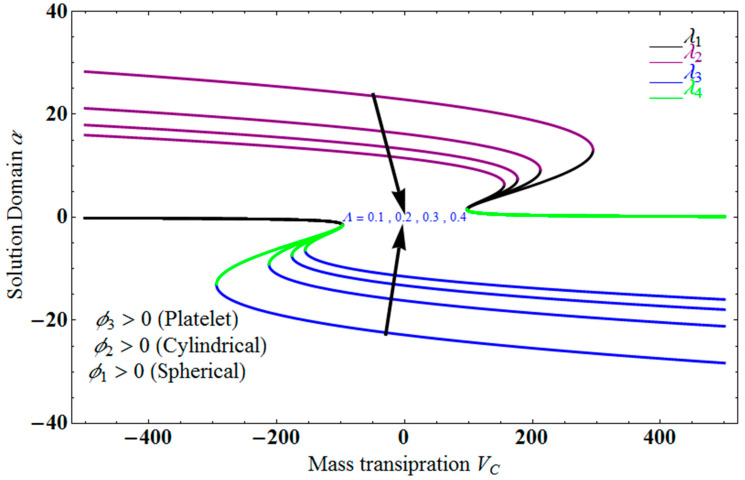
Impact of solution domain α verses mass transpiration $V_{c}$ for varying couple stress Λ.

**Figure 3:**
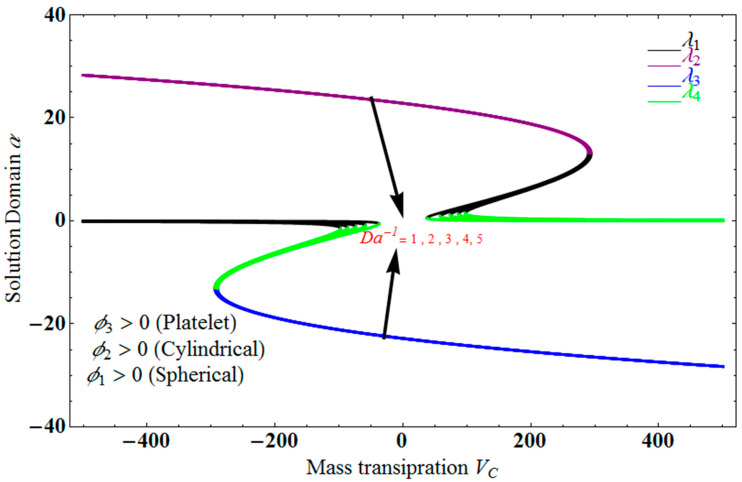
Impact of solution domain α verses mass transpiration $V_{c}$ for varying $Da^{-1}$.

Figure 2 depict the impact of solution domain α on mass transpiration $V_{c}$ with various values of couple stress parameters for the stretching sheet. In this graph, we can see the results of four roots $\alpha_{1},\;\alpha_{2},\;\alpha_{3}\;\text{and}\;\alpha_{4}$. $\alpha_{1},\;\alpha_{2}$ are were found on the *x*-axis, whereas $\alpha_{3},\;\alpha_{4}$ were on the positive *y*-axis. These four roots are completely contrary to one another. For the roots $\alpha_{1},\;\text{and}\;\alpha_{4}$, it can be seen that the solution domain is greater for higher values of couple stress. However, when the couple stress parameter values increase in the roots $\alpha_{2},\;\text{and}\;\alpha_{3}$, then the solution domain decreases. The results of a modeling of dispersion water with three different types of nanoparticles are water-Al_2_O_3_/single wall carbon nanotubes/graphene when $Pr\;=6.27\;\;\;\text{and}\;\;\phi_{1}=\phi_{2}=\phi_{3}=0.1$ and the other parameters remain constant. Similar effects apply in Figure 3 for different parameters, such as the inverse Darcy number. 

**Figure 4:**
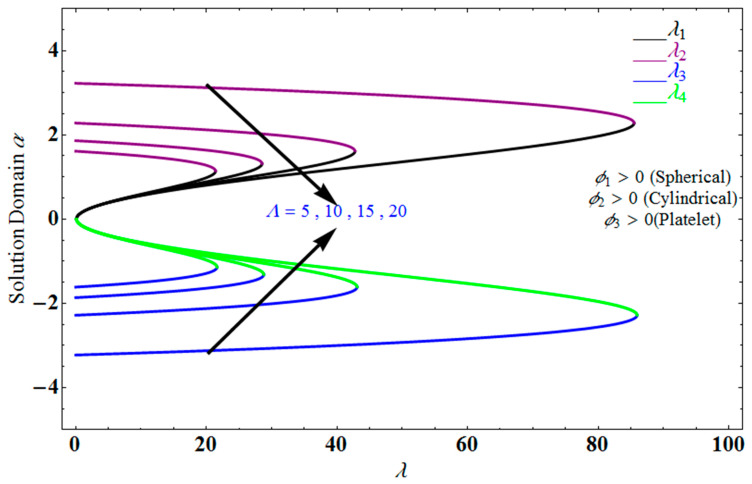
Impact of solution domain α verses λ for varying Λ.

**Figure 5:**
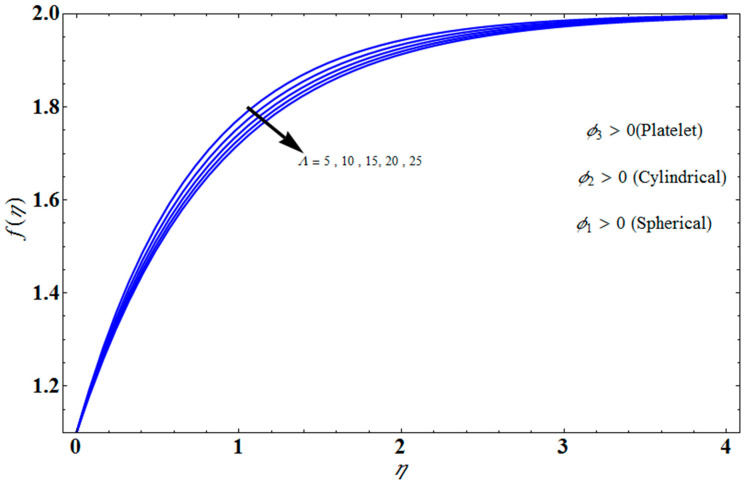
Influence of f(η) on η for several values of Λ.

**Figure 6:**
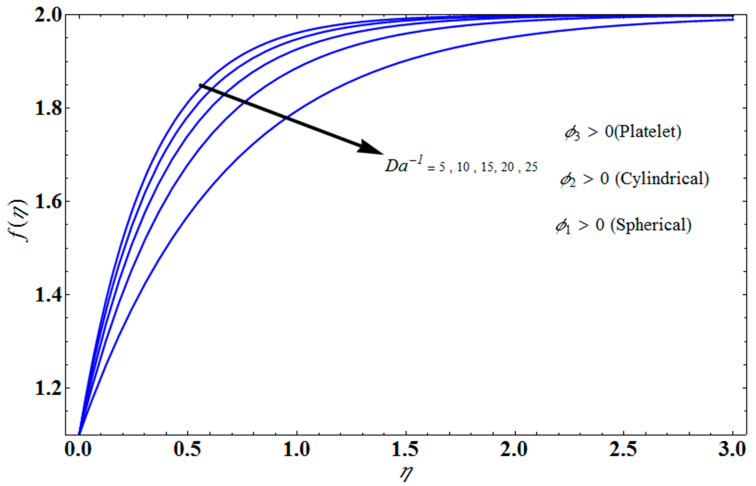
Effect of f(η) on η for several values of $Da^{-1}$.

**Figure 7:**
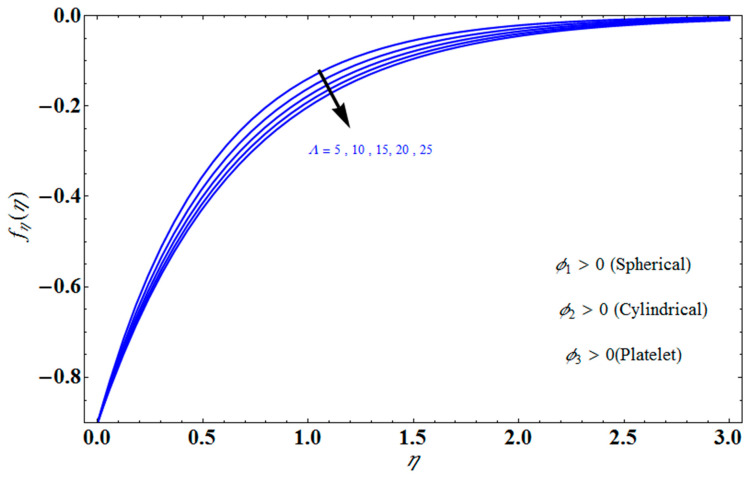
Influence of $f_{\eta }\left(\eta \right)$ on η for several values of Λ.

**Figure 8:**
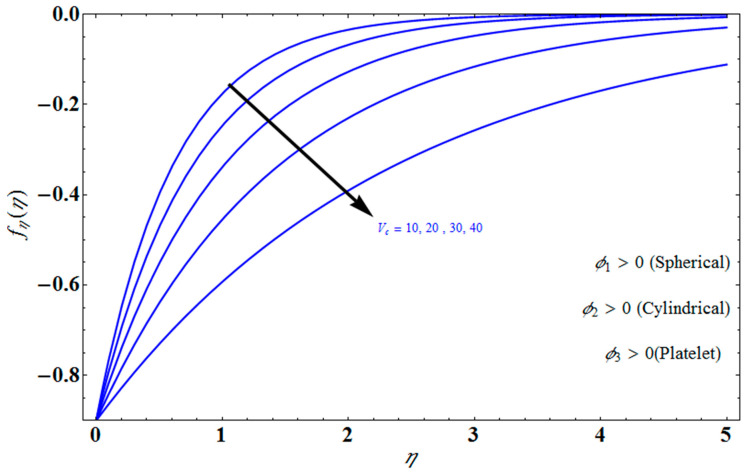
Influence of $f_{\eta }\left(\eta \right)$ on η for several values of $V_{c}$.

**Figure 9:**
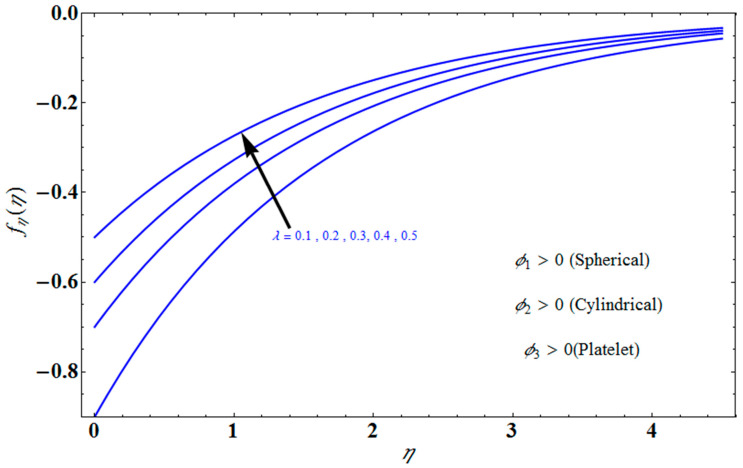
Effect of $f_{\eta }\left(\eta \right)$ on η for different values of λ.

**Figure 10:**
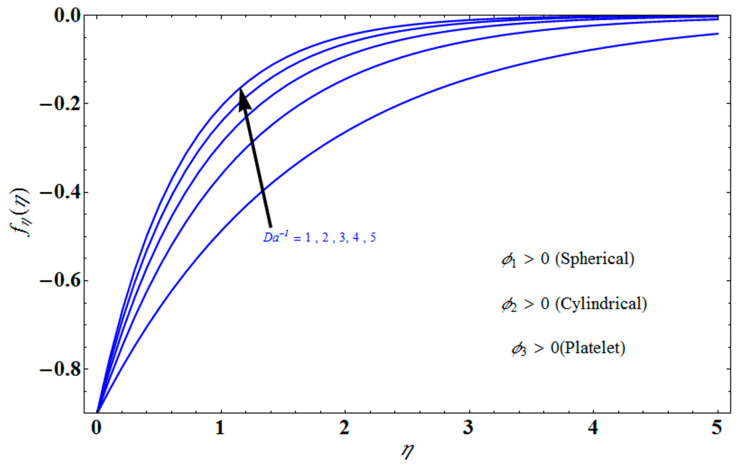
Effect of $f_{\eta }\left(\eta \right)$ on η for different values of $Da^{-1}$.

Figure 4 depicts the impact of solution domain α on λ with various values of the couple stress parameter. In this graph, we can see the results of the four roots $\alpha_{1},\;\alpha_{2},\;\alpha_{3}\;\text{and}\;\alpha_{4}$. $\alpha_{1},\;\alpha_{2}$ were found on the *x*-axis, whereas $\alpha_{3},\;\alpha_{4}$ were on the positive *y*-axis. These four roots are completely contrary to one another. For the roots $\alpha_{1},\;\text{and}\;\alpha_{4}$ it can be seen that solution domain is greater for higher values of couple stress. However, when the couple stress parameter values increase in the roots $\alpha_{2},\;\text{and}\;\alpha_{3}$ then the solution domain decrease. Figure 5 represents the effect of f(η) on η for different values of the couple stress parameter Λ for the stretching surface, keeping other parameters constant. When couple stress parameter Λ increases, the thermal boundary decreases. In fact, the force and impact of the boundary conditions override the couple stress parameter Λ in the case of stretching sheets. An increase in the value of the couple stress parameter Λ causes a comparable rise in the axial velocity profiles in the case of both porous stretching and shrinking. The boundary layer becomes thinner when non-Newtonian viscoelastic shear stress occurs. Additionally, the boundary layer is smaller when suction, as opposed to injection, is utilized. Figure 6 represents the effect of f(η) on η for different values of the inverse Darcy number for stretching surface, keeping the other parameters constant. When the Darcy number increases, the thermal boundary decreases. The inverse Darcy number, as previously stated, is an attractive body force whose projection on the *x*-axis is situated in the opposite direction of *x*. Therefore, a higher inverse Darcy number value will result in more resistance to the axial velocity, thus reducing it. On the other hand, for a stretching sheet, the magnetic field and the imposed boundary condition are both in the same direction and will reinforce one another. Figure 7 represents the effect of $f_{\eta }\left(\eta \right)$ on η for different values of couple stress parameter Λ for the stretching surface, keeping other parameters constant. When couple stress parameter Λ increases, the thermal boundary decreases. Figure 8 represents the effect of *f_η_*(*η*) on η for different values of mass transpiration $V_{c}$ for the stretching surface, keeping other parameters constant. When mass transpiration $V_{c}$ increases, the thermal boundary decreases. The noticed increase in influence is due to the dimensionless number’s simple index of the ratio of thermal resistances within the fluid, and at its surface, the mass transpiration is only significant close to the wall. Figure 9 represents the effect of $f_{\eta }\left(\eta \right)$ on η for different values of λ for the stretching surface, keeping the other parameters constant. When λ increases, thermal boundary increases. Figure 10 represents the effect of $f_{\eta }\left(\eta \right)$ on η for different values of the Darcy number for the stretching surface, keeping the other parameters constant. When the Darcy number increases, the thermal boundary increases. In fact, the BC’s power and influence are more significant while stretching a sheet, unlike the results for the fluids. Similar results are obtained by increasing the size of the Darcy number. Axial velocity profiles in the case of both mass transpiration parameters and stretching/shrinking increase. The dividing mass transpiration in the stretching/shrinking sheet causes the layer to narrow.

**Figure 11:**
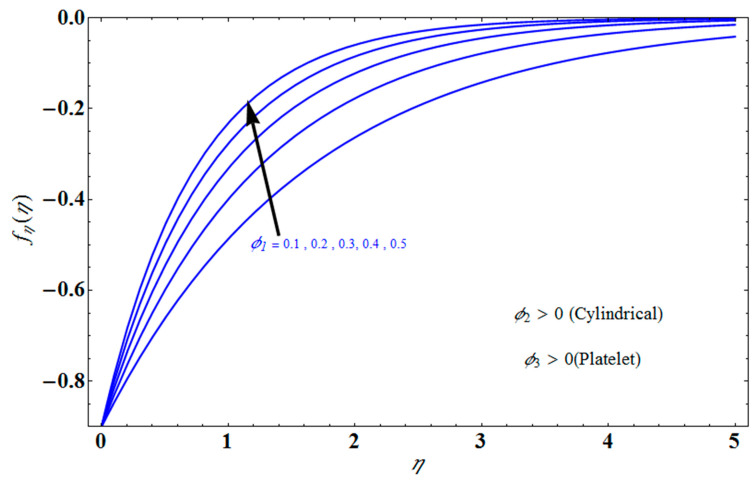
Influence of $f_{\eta }\left(\eta \right)$ on η for different values of $\phi_{1}$.

**Figure 12:**
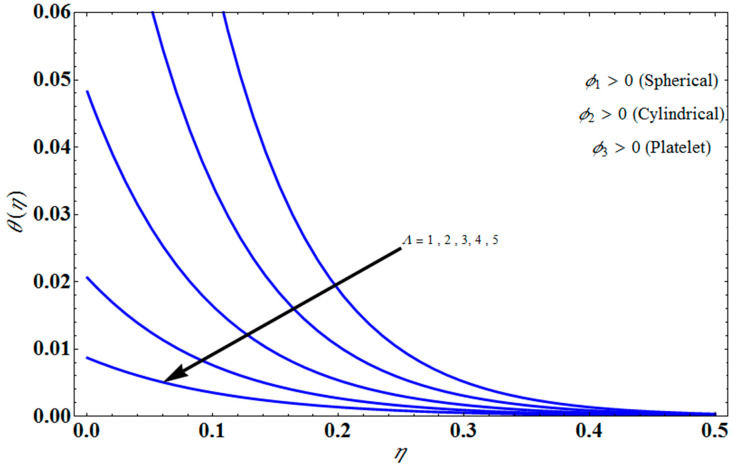
Effect of θ(η) on η for different values of Λ.

**Figure 13:**
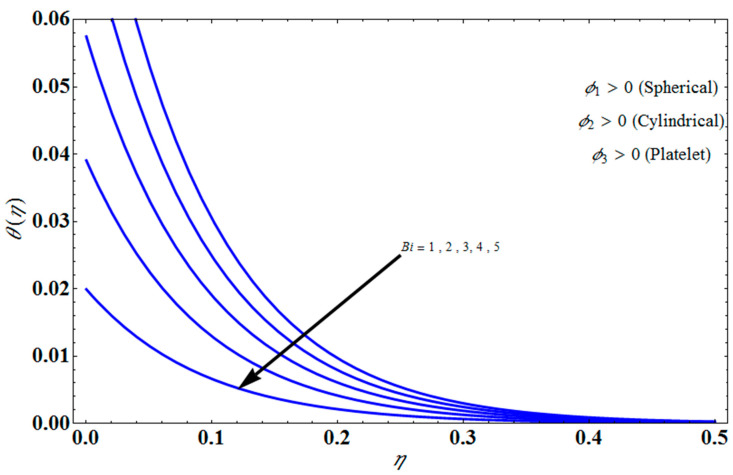
Influence of θ(η) on η for different values of Bi.

**Figure 14:**
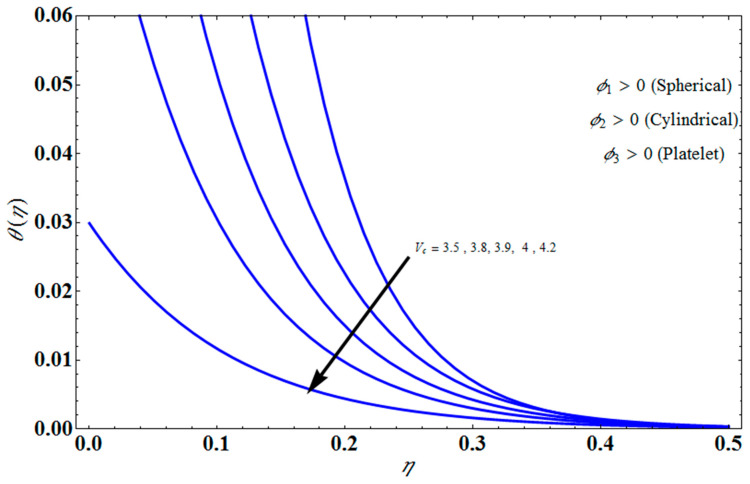
Influence of θ(η) on η for different values of $V_{c}$.

**Figure 15:**
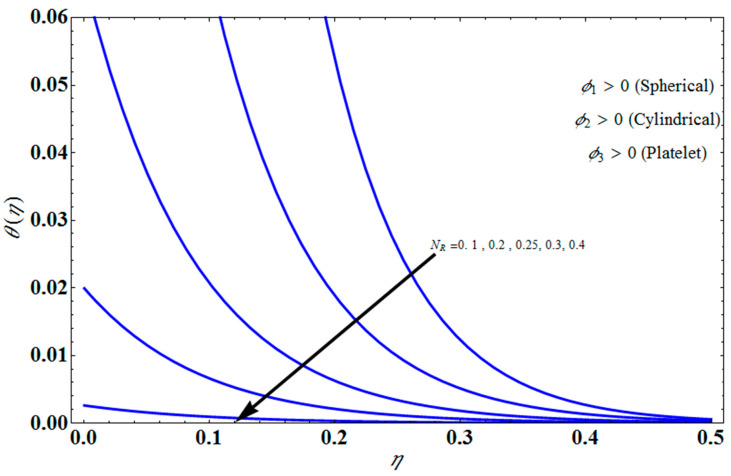
Influence of θ(η) on η for several values of $N_{R}$.

**Figure 16:**
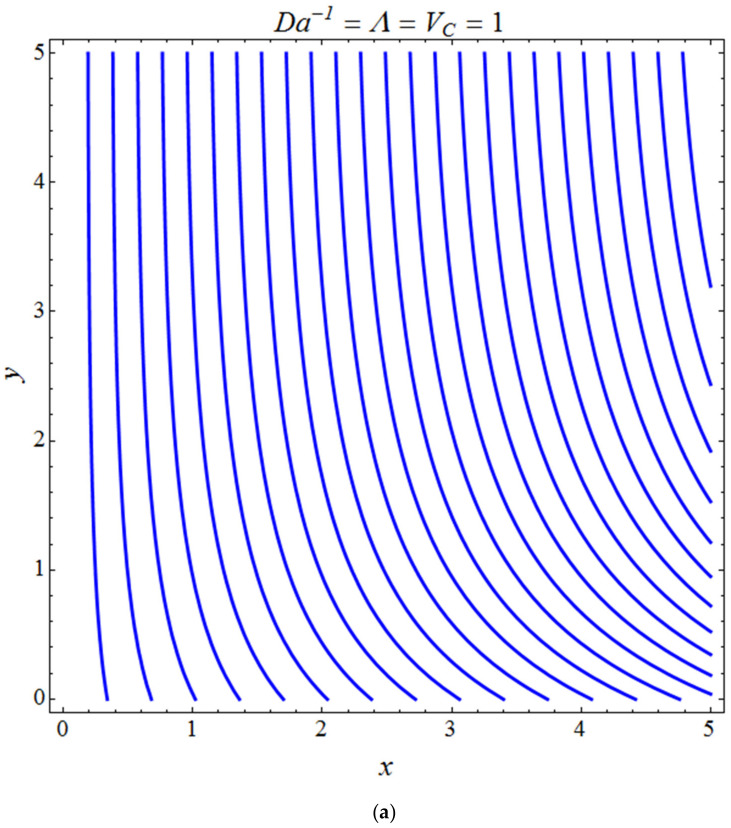

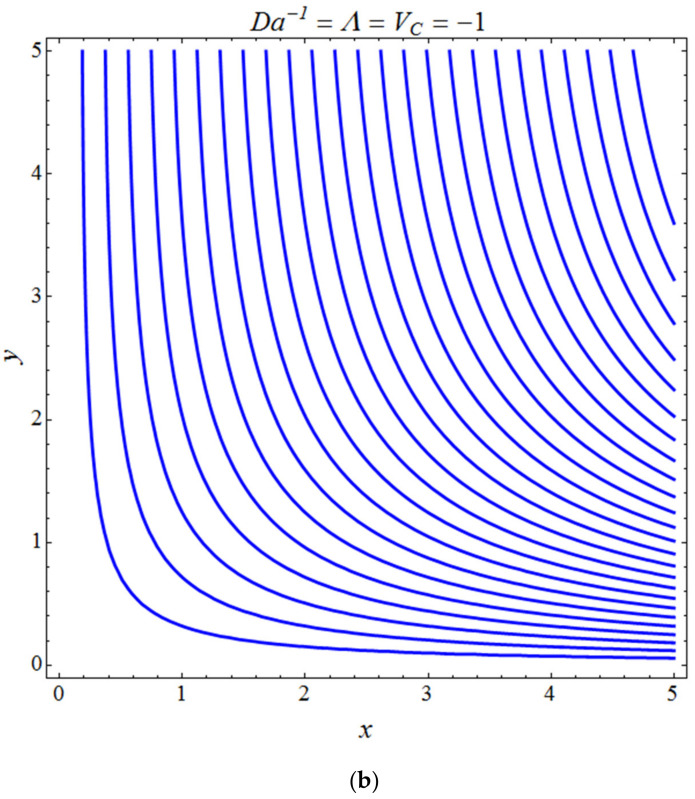
Pattern of streamline flows for (**a**) suction and (**b**) injection cases.

**Figure 17:**
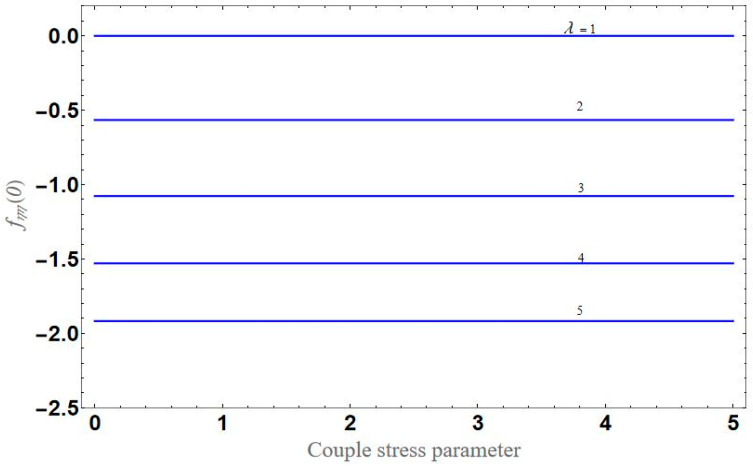
Variation of CfRex1/2 with respect to couple stress for different values of λ.

**Figure 18:**
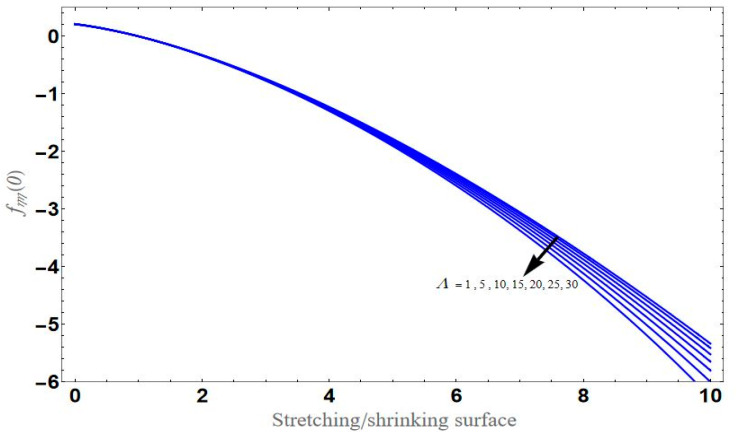
Variation of CfRex1/2 with respect to the stretching/shrinking surface for different values of couple stress Λ.

Figure 11 represents the effect of $f_{\eta }\left(\eta \right)$ on η for different values of volume fraction $\phi_{1}$ for the stretching surface, keeping the other parameters constant. When $\phi_{1}$ increases, the thermal boundary increases. The ternary hybrid nanoparticles within the nanofluid increase as the volume fraction of the nanoparticles increases, allowing for an increased area for improved heat conduction. As can be seen in Figure 11, this raises the velocity of the nanofluid. Figure 12 shows the impact of θ(η) on η for various values of Λ for the stretching surface. θ(η) decreases with an increase the value of Λ, and it is observed that the boundary layer thickness also reduces in the stretching surface. Figure 13 indicates the impact of θ(η) on η for various values of the Biot number for the stretching case. Here, it seen that θ(η) decreases as the value of the Biot number increases. As a result, the Biot number enhances the thermal diffusivity of a nanofluid for rising quantities of *Bi*. The nanofluids are increase in the efficiency of the *Bi* as the temperature rises. The performance of nanofluids is affected by both the drop in particle volume fraction and the increase in temperature in the flow pattern. Figure 14 show the temperature distribution θ(η) versus the similarity variable η for various values of the mass transpiration *V_c_* for the stretching surface. This means that the thickness of the thermal boundary layer under suction is thinner than under blowing. Figure 15 show the temperature distribution θ(η) versus the similarity variable η for various values of the thermal radiation *N_R_* for the stretching surface. Thermal radiation, therefore, increases the thermal diffusivity of nanofluids; for emerging radiation parameter values, heat will be added to the regime, and temperatures will rise as a result. A fluid temperature greater than both the wall temperature and the surrounding ambient temperature is technically possible, as was mentioned for the heat transmission of flows over a stretching sheet. Here, we have discussed the effect of forced flow over a stretching sheet; we now look at heat transport in the presence of radiation. The effect of heat conductivity is amplified by the radiation. Radiation has the effect of dampening or enhancing heat transmission in a linear manner. Figure 16a,b show the streamline flow patterns for suction and injection situations, respectively. For suction and injection scenarios, we look at different line patterns. The streamline flow pattern for the stretched boundaries is shown in Figure 16a,b for varied levels of mass transpiration *V_c_* while other parameters remain constant. When wall suction occurs at specific locations, as predicted by the physical theory, the flow field becomes regularized. On the other hand, blowing (flow injection) eliminates the streamline in the circular shape. Figure 17 indicates the effect of the skin friction coefficient on the stretching/shrinking surface λ as a function of couple stress Λ, respectively. Here, it is noticed that the couple stress parameter is the reciprocal of thickness decreasing of the boundary layer occurs via the same process. While skin friction is linearly proportional to the thickness of the boundary layer, it is estimated to have an inverse proportionality λ, as depicted in Figure 17. In addition, upward pulling couple stress parameters will draw fluid toward the negative *y* direction and reduce skin friction. The thickness of the boundary layer will decrease when the suction parameter, which measures the strength of wall suction, is increased, and as a result, the gradient of the velocity on the wall will also increase. This impact will enhance skin friction, as shown in Figure 17. Figure 18 shows the effect of the skin friction coefficient on couple stress Λ as a function of the stretching/shrinking surface λ, respectively. Here, it should be noted that the process of the decrease of the boundary layer’s thickness and the stretching/shrinking surface parameter are the same. Skin friction is thought to have an inverse relationship with boundary layer thickness, as shown in Figure 18, even though the two are linearly proportional λ. Additionally, fluid will be drawn toward the negative *y* direction, and the skin friction will be decreased by the upward pulling couple stress parameters. The suction parameter, which gauges the strength of the wall suction, causes a reduction in the thickness of the boundary layer, which causes an increase in the gradient of velocity on the wall. The increase in skin friction caused by this collision is depicted in Figure 18.

## 4. Conclusions

The present study examines a ternary hybrid nanofluid flow towards a porous stretching surface using water as the base fluid. The current problem’s ODEs were derived by controlling PDEs with similar variables that were solved analytically to provide the solution domain. The solution domain can be used to investigate the solution domain, axial velocity, and heat transfer. Some comparisons of our results are given in Table 2. The results of the current effort can then be analyzed using various physical characteristics and graphical arrangements. 

The values of $\alpha_{1}$ and $\alpha_{2}$ increase as the value of Λ increases, while the values of $\alpha_{3}$ and $\alpha_{4}$ decrease as the value of Λ for the stretching condition is increased.

The values of $\alpha_{1}$ and $\alpha_{2}$ increase as the value of λ increases, while the values of $\alpha_{3}$ and $\alpha_{4}$ decrease as the value of λ is increased.

f(η) decreases when the value of Λ and $Da^{-1}$ increase.

$f_{\eta }\left(\eta \right)$ decreases when the value of Λ and $V_{c}$ increase, but $f_{\eta }\left(\eta \right)$ increases when the value of $Da^{-1}$.

θ(η) decreases when the value of $\Lambda ,\;Bi,\;V_{c},\;\text{and}\;N_{R}$ increase, for the stretching case.

## Figures and Tables

**Table 1:** Thermophysical properties of different nanoparticles.

	Thermophysical Properties	Base Fluid	Aluminum Oxide (Al_2_O_3_)	Single-Wall *CNT*	Graphene (*G*)	Shape
1	$\rho \left(\mathrm{kg}/m^{3}\right)$	997.1	3970	8933	2200	Spherical
2	$C_{p}\left(J/\mathrm{kg}\;K\right)$	4179	765	385	790	Cylindrical
3	κ(W/m K)	0.613	40	401	5000	Platelet

**Table 2:** Comparing the results.

Related Works by Other Authors	Fluids	Methods	Momentum Equation
Manjunatha et al. [14], Theoretical Study of Convective Heat Transfer in Ternary Nanofluid Flowing past a Stretching Sheet	Non-Newtonian	Numerical method	$u\;u_{x}+\;v\;u_{y}\;\;=\;\nu_{thnf}\;u_{yy}+\frac{\sigma_{thnf}B_{0}^{2}}{\rho_{thnf}}\;\;u,$ MHD, ternary hybrid nanofluid with stretching sheet
Enran et al. [15], Dynamics of Tri-Hybrid Nanoparticles in the Rheology of Pseudo-Plastic Liquid with Dufour and Soret Effects	Non-Newtonian	Numerical method	u∂u∂x +v∂u∂y= μthnfρthnf ∂∂x(|∂u∂y|m−1∂u∂y) −μthnfρthnfKFDu−FD(ks)12u2+gα(T−T∞)+gβ(C−C∞), porous medium with mixed convection
Saleem et al. [16], Insight into the Motion of Water Conveying Three Kinds of Nanoparticles on a Horizontal Surface: Significance of Thermo-Migration and Brownian Motion of Different Nanoparticles	Non-Newtonian	Numerical method	$u\;u_{x}+\;v\;u_{y}\;\;=\;\nu_{thnf}\;u_{yy}$
present work	Non-Newtonian	Analytical method	$\begin{array}{l} \rho_{tnf}\left(\frac{\partial u}{\partial t}+u\frac{\partial u}{\partial x}+v\frac{\partial u}{\partial y}\right)=-\frac{\partial P}{\partial x}\\ +\mu_{tnf}\left(\frac{\partial^{2}u}{\partial x^{2}}+\frac{\partial^{2}u}{\partial y^{2}}\right)-\frac{\mu_{tnf}}{K}\left(u_{\infty }-u\right)-\eta_{0}\frac{\partial^{4}u}{\partial y^{4}} \end{array}$ couple stress of fluid, unsteady case, porous medium, and ternary hybrid nanofluid

## Nomenclature

**Symboles**	**Descriptions**	**S. I. Units**
$A_{1}\;\mathrm{to}\;A_{4}$	ternary nanofluid constants	(-)
$B_{1}\;\mathrm{to}\;B_{4}$	constants	(-)
Bi	Biot number	(-)
*Da^−1^*	inverse Darcy number	(-)
$f_{\eta }\left(\eta \right)$	axial velocity	(-)
F(η)	f(η)+η transverse velocity	(-)
G(t,x)	integration constant	(-)
h	coefficient of heat transfer	(-)
*K*	permeability	(-)
$k^{*}$	mean absorption coefficient	(m^−2^)
*N_R_*	radiation parameter	(-)
*P*	pressure	(Nm^−2^)
*Pr*	Prandtl number	(-)
$q_{r}$	heat flux	(Wm^−2^)
*T*	temperature	(K)
$T_{w}$	temperature at wall	(K)
$T_{\infty }$	temperature from field	(K)
*t*	time	(s)
$U_{w}$	velocity at wall	(ms^−1^)
$U_{\infty }$	unsteady free stream velocity	(ms^−1^)
$U_{0}$	constant	(-)
(u, v)	velocities along *x* and *y* direction	(ms^−1^)
$V_{w}$	wall transpiration velocity	(ms^−1^)
$V_{c}$	mass transpiration	(-)
*Greek Symbols*		
α	solution domain	(-)
β	unsteadiness parameter	(-)
γ	constant	(-)
$\eta_{0}$	materials for the couple stress fluid	(-)
$\phi_{1},\phi_{2},\;\;\text{and}\;\;\phi_{3}$	solid volume fractions for three different nanoparticles	(-)
λ	stretching/shrinking sheet	(-)
Λ	couple stress parameter	(-)
μ	dynamic viscosity of fluid	(Kg m^−1^ s^−1^)
κ	thermal conductivity	(m^2^s^−1^)
ν	kinematic viscosity	(m^2^s^−1^)
θ(η)	temperature profile	(-)
$\sigma^{*}$	Stefan–Boltzmann constant	(W m^−2^K^−4^)
ρ	density	(Kg m^−3^)
Γ	incomplete gamma function	(-)
ψ(x,y,t)	stream function	(-)
*Subscripts*		
w	wall	(-)
∞	far away from the sheet	(-)
f	base fluid	(-)
tnf	ternary hybrid nanofluid	(-)
*Abbreviations*		
B. Cs	boundary conditions	(-)
ODE	ordinary differential equations	(-)
PDE	partial differential equations	(-)
